# The power of parallel structure

**DOI:** 10.1007/s40037-015-0227-3

**Published:** 2015-10-19

**Authors:** Chris Watling

**Affiliations:** Departments of Clinical Neurological Sciences and Oncology, Schulich School of Medicine and Dentistry, Western University, London, ON Canada

In the writer’s craft section we offer simple tips to improve your writing in one of three areas: Energy, Clarity and Persuasiveness. Each entry focuses on a key writing feature or strategy, illustrates how it commonly goes wrong, teaches the grammatical underpinnings necessary to understand it and offers suggestions to wield it effectively. We encourage readers to share comments on or suggestions for this section on Twitter, using the hashtag: #how’syourwriting?

Exploiting opportunities to create parallel structure is a surefire technique to improve your writing. Parallel structure simply means that ideas that are similar in content or function are expressed in a similar way [1]. To present a pair or a series of ideas using parallel structure, each idea should be written in a similar grammatical and stylistic form. Parallel constructions—within sentences or paragraphs—guide readers elegantly through your story. When we use parallel constructions, we *show* our readers how our ideas are linked, rather than forcing them to work to see the connections.

Orators have long understood the power of parallel structure. Consider this paragraph from Martin Luther King Jr.’s ‘I have a dream’ speech:

Now is the time to make real the promises of democracy. Now is the time to rise from the dark and desolate valley of segregation to the sunlit path of racial justice. Now is the time to lift our nation from the quicksands of racial injustice to the solid rock of brotherhood. Now is the time to make justice a reality for all of God's children. [2]

This iconic speech stands not only as a masterpiece of oratory, but also as a masterpiece of parallel construction. The paragraph itself is a parallel construction, with its four sentences each leading off with the invocation ‘Now is the time.’ And within each of the two middle sentences, evocative contrasts are presented in parallel form.

Using parallel structure effectively is not the sole purview of master orators, however. Once you know where to look for opportunities, you can use parallel structure to enliven your own writing. Within sentences, there are two key opportunities. First, look for situations where you enumerate a series of ideas, and then ensure that each item in the series is similarly constructed. A series might comprise short phrases separated by commas or semicolons, or even set off by numbers. Let’s consider an example:

*Faculty members juggled a number of competing responsibilities, including to provide feedback, assessment of learners, and developing curricula*.

In this series, the three competing responsibilities are expressed in three different ways: as an infinitive (‘to provide’), as a noun (‘assessment’), and as a gerund (‘developing’). The effect of this verbal eclecticism is that the writing loses momentum, and the reader gets stuck mid-sentence. If each idea were expressed in a similar fashion, the sentence would flow much more easily:

*Faculty members juggled a number of competing responsibilities, including providing feedback, assessing learners, and developing curricula*.

Here each task is expressed as a gerund (an ‘-ing’ verb used as a noun), but there is more than one way to bring parallel structure to this sentence. Each task could also be expressed as a noun:

*Faculty members juggled a number of competing responsibilities, including feedback provision, learner assessment, and curriculum development*.

The key is to be consistent within a series.

Second, look for opportunities to use joining words to link two ideas within the same sentence. Commonly used joining words include:

Not only…but also

Both…and

Either…or

Neither…nor

The trick to making joining words work for you is to ensure that the words that follow the first word or phrase are similar in form to those that follow the second [3]:

Stroke units not only **reduce** mortality, but also **improve** functional outcomes.

In this sentence, the word that follows *not only* (‘reduce’) is the same form—a present tense verb—as the word that follows *but also* (‘improve’), which not only creates a pleasing symmetry, but also reinforces the connection between the two ideas.

Just as parallel structure strengthens sentences, so it breathes life into paragraphs. As key building blocks of any manuscript, paragraphs elaborate ideas, advance logic, and build arguments. Consider the following short paragraph about the state of the science on feedback:

Considerable attention has been paid to the structural aspects of feedback, leading to articles and workshops aimed at educating feedback providers about how to construct and deploy feedback effectively. The issue of learners’ responses to feedback has received less attention, although a growing body of literature has been exploring this area. We have not undertaken a critical examination of medicine’s learning culture and how it might enable or constrain the exchange of meaningful feedback.

The paragraph is grammatically correct, but lacks punch and style. The solution? Create a parallel structure to link the paragraph’s three central ideas:

*We have paid considerable attention* to the structural aspects of feedback, leading to articles and workshops aimed at educating feedback providers about how to construct and deploy feedback effectively. *We have devoted less attention* to learners’ responses to feedback, although a growing body of literature has been exploring this area. *And we have virtually ignored* medicine’s learning culture and how it might enable or constrain the exchange of meaningful feedback.

Of course, one rarely uses a single writing technique to solve a style problem. Here, I have also used active rather than passive verb constructions for the first two ideas, and I have strengthened the third verb phrase from ‘we have not undertaken’ to ‘we have virtually ignored.’

Next time you are editing your own work, challenge yourself to create parallel constructions wherever possible. The result will be stronger, clearer, and more persuasive prose.
